# Identification of Potential Prognostic Biomarkers Associated With Cancerometastasis in Skin Cutaneous Melanoma

**DOI:** 10.3389/fgene.2021.687979

**Published:** 2021-07-21

**Authors:** Yang Li, Shanshan Lyu, Zhe Gao, Weifeng Zha, Ping Wang, Yunyun Shan, Jianzhong He, Suyang Huang

**Affiliations:** ^1^Dermatology, The Third People’s Hospital of Hangzhou, Hangzhou, China; ^2^Department of Pathology, Guangdong Provincial People’s Hospital, Guangdong Academy of Medical Sciences, Guangzhou, China; ^3^Department of Pathology, The Fifth Affiliated Hospital, Sun Yat-sen University, Zhuhai, China

**Keywords:** skin cutaneous melanoma, immune microenvironment, WGCNA, metastasis, prognostic biomarkers

## Abstract

Skin cutaneous melanoma (SKCM) is a highly aggressive tumor. The mortality and drug resistance among it are high. Thus, exploring predictive biomarkers for prognosis has become a priority. We aimed to find immune cell-based biomarkers for survival prediction. Here 321 genes were differentially expressed in immune-related groups after ESTIMATE analysis and differential analysis. Two hundred nineteen of them were associated with the metastasis of SKCM *via* weighted gene co-expression network analysis. Twenty-six genes in this module were hub genes. Twelve of the 26 genes were related to overall survival in SKCM patients. After a multivariable Cox regression analysis, we obtained six of these genes (PLA2G2D, IKZF3, MS4A1, ZC3H12D, FCRL3, and P2RY10) that were independent prognostic signatures, and a survival model of them performed excellent predictive efficacy. The results revealed several essential genes that may act as significant prognostic factors of SKCM, which could deepen our understanding of the metastatic mechanisms and improve cancer treatment.

## Introduction

Skin cutaneous melanoma (SKCM) is a high-mortality-rate malignant tumor caused by abnormal melanocyte proliferation in neural crest cells (Bray et al., 2018; Siegel et al., 2020). According to the GLOBOCAN database (gco.iarc.fr), there were more than 200,000 new cases of SKCM over the world, and a quarter of them died in 2018 (Bray et al., 2018). The leading cause of death from this cancer is the metastasis of multiple organs (Zhu et al., 2016). The mortality rate of SKCM patients was significantly higher than that of other malignant tumors (Ekwueme et al., 2011). Therefore, SKCM seriously threatens public health and has become one of the evilest tumors worldwide (Gershenwald et al., 2017). The risk factors of SKCM included atypical mole or dysplastic nevus patterns and increased mole count (Chen et al., 2013). The treatment of the tumor microenvironment (TME) as a new treatment strategy has attracted public attention (Yang et al., 2018). It is composed of numerous cell types and is involved in the occurrence and invasion of tumors (Hanahan and Weinberg, 2000). With the development of tumor cytology and molecular biology, a deeper understanding of TME is essential to reveal improved immunotherapy (Li et al., 2017; Qian et al., 2018). An algorithm called ESTIMATE could estimate the abundance of immune cells according to the gene expression level of tissues (Yoshihara et al., 2013; Li et al., 2016). Research shows that targeting stromal cells and connective tissue cells can be a new way to overcome drug resistance effectively (Hemminki et al., 2020).

Weighted gene co-expression network analysis (WGCNA) is a computational method often used to explore the relationship between genes and clinical characteristics (Langfelder and Horvath, 2008; Yuan et al., 2020). The significant dominance of WGCNA is to combine genes into co-expression modules and build the relationship between clinical traits and genes (Luo et al., 2019). WGCNA could analyze a mass of genes and identify expression modules related to clinical features and critical genes for further verification (Luo et al., 2019; Radulescu et al., 2020).

**FIGURE 1:**
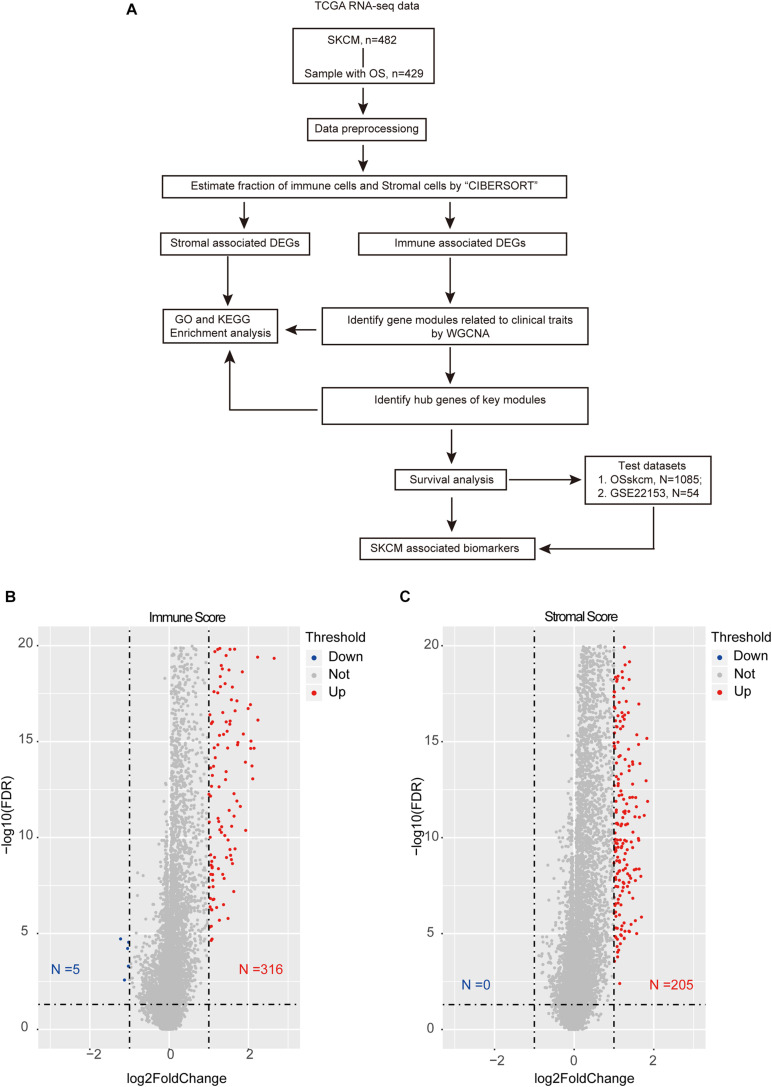
Overview of the integration analysis. **(A)** Workflow of the analysis. **(B)** Volcano plot showing the differentially expressed genes (DEGs) between high- and low-immune-score samples. **(C)** Volcano plot showing the DEGs in high and low stromal score groups. The red color indicates the up-regulated genes, while blue represents the down-regulated ones. The horizontal dotted line represents a false discovery rate equal to 0.05, and the vertical dotted line represents a fold change equal to 2 or 0.5.

This study obtained several modules and hub genes with significant differences in tumor microenvironment based on WGCNA and identified potential biomarkers that can predict SKCM prognosis (Figure 1A).

## Materials and Methods

### Data Sources

Any ethical issue did not involve this study because it used public data which has already been published. We extracted the expression matrix of 473 SKCM patients and their clinical information from TGGA. Only 429 SKCM patients with complete overall survival information were selected. The clinical information of these patients (including gender, weight, pathologic stage, and so on) are shown in Table 1 and Supplementary Table 1. The gene expression profiles were quantified by fragments per kilobase of transcript per million mapped reads and normalized through log2-based transformation. Besides that, the immune and stromal scores of each sample were calculated by the ESTIMATE analysis. The high-immune-score group represented the high proportion of immune cells in the tumor microenvironment, and the low-immune-score group represented conversely. The stromal score plays the same role but represents the stromal cell. An independent test dataset that contains 54 SKCM patients was downloaded from the Gene Expression Omnibus database.

**TABLE 1:** Clinicopathological characteristics of 429 skin cutaneous melanoma patients in The Cancer Genome Atlas dataset.

Clinical and pathological indices	Case no.	OS (%)	*P*-value^*a*^
Specimens	429		
Mean age	58		
Age (years)			<0.001
≤58	216	52.8	
>58	213	54.5	
Gender			0.135
Male	266	49.2	
Female	163	60.7	
pTNM stage			<0.001
I	114	47.4	
II	127	55.9	
III	166	56.0	
IV	22	54.5	
Sample type			<0.001
Metastatic	349	48.9	
Primary	80	73.8	

*^a^Log-rank test using the Kaplan–Meier method. P < 0.05 was considered significant. OS, overall survival.*

### Differential Analysis

The patients were classified into high- or low-immune-score groups and stromal score groups based on the median score of the ESTIMATE analysis. Then, differential analyses were used to filter the differentially expressed genes (DEGs) between the high and low groups. Finally, the raw *P*-value was corrected by false discovery rate (FDR). The differential analysis was performed through the “limma” R package, and the threshold was FDR < 0.05 and | log2 FC| ≥ 1 (Supplementary Table 2).

### Constructed WGCNA Network and Identified Modules

We performed WGCNA analysis on the immune-related DEGs by the “WGCNA” R package. First, the *pickSoftThreshold* function was used to select the soft threshold (power) to construct the non-scale network. In this study, the power was set at 10. Second, modules were detected by the hierarchical clustering function “blockwiseModules.” Then, the modules were associated with clinical characteristics by calculating gene significance (GS) and module membership (MM). Although a correlation between traits and modules has been found and the most relevant modules can be selected for analysis, the modules themselves still contain a large number of genes, so it is necessary to further search for the most important genes. All modules can be correlated with genes, and all continuous traits can also be correlated with gene expression levels. If genes significantly associated with traits are also significantly associated with a particular module, then those genes are likely to be crucial. Finally, the crucial genes in the candidate modules were filtered for further analysis. The cutoff for screening important genes was GS > 0.25 and MM > 0.8 (Liang et al., 2020).

### Enrichment Analysis

All the DEGs and candidate genes were subjected to an enrichment analysis using the “clusterProfile” R package (Yu et al., 2012). The functional background datasets contained the Gene Ontology (GO) terms (Dennis et al., 2003) and Kyoto Encyclopedia of Genes and Genomes (KEGG) pathways (Kanehisa et al., 2017). Functions with a FDR < 0.05 were selected for further discussion.

### Validation of Candidate Genes

GEPIA^1^ was used to validate the immune-related DEGs. The web server collected the expression data of 9,736 tumor patients and 8,587 normal samples from The Cancer Genome Atlas (TCGA) and the GTEx projects. For the transcriptional level validation in SKCM, we set the criteria of significant results to | log2 FC| ≥ 0.585 and *P* < 0.05. We used the TIMER web server to verify whether the crucial genes are associated with the immune cell infiltrate levels.

### Survival Analysis

Survival analysis was used to filter vital prognostic biomarkers through the “survival” R package. The signature was filtered as independent of other clinical features through multivariable Cox regression analysis. Then, the independent clinical genes were used to combine a new survival signature by the Cox regression model. The risk score was calculated by the expression of selected crucial genes, and correlation was estimated by Cox regression coefficients through the following formula:

Riskscore=(expgene1coef*gene1)+(expgene2coef*gene2)+…+(expgeneNcoef*geneN)

Then, we performed an area under the receiver operating characteristics (ROC) curve index to explore the prognostic efficiency of this signature using the “pROC” R package. The OSskcm Tool, which combined the survival information of more than 1,000 SKCM patients, was used to test the prognostic ability of the candidate genes (Zhang et al., 2020). An independent test dataset that contains 54 SKCM patients was used to verify the prognostic efficacy of the survival model (GSE22153).

## Results

### Identification of Immune- and Stromal-Associated DEGs

**FIGURE 2:**
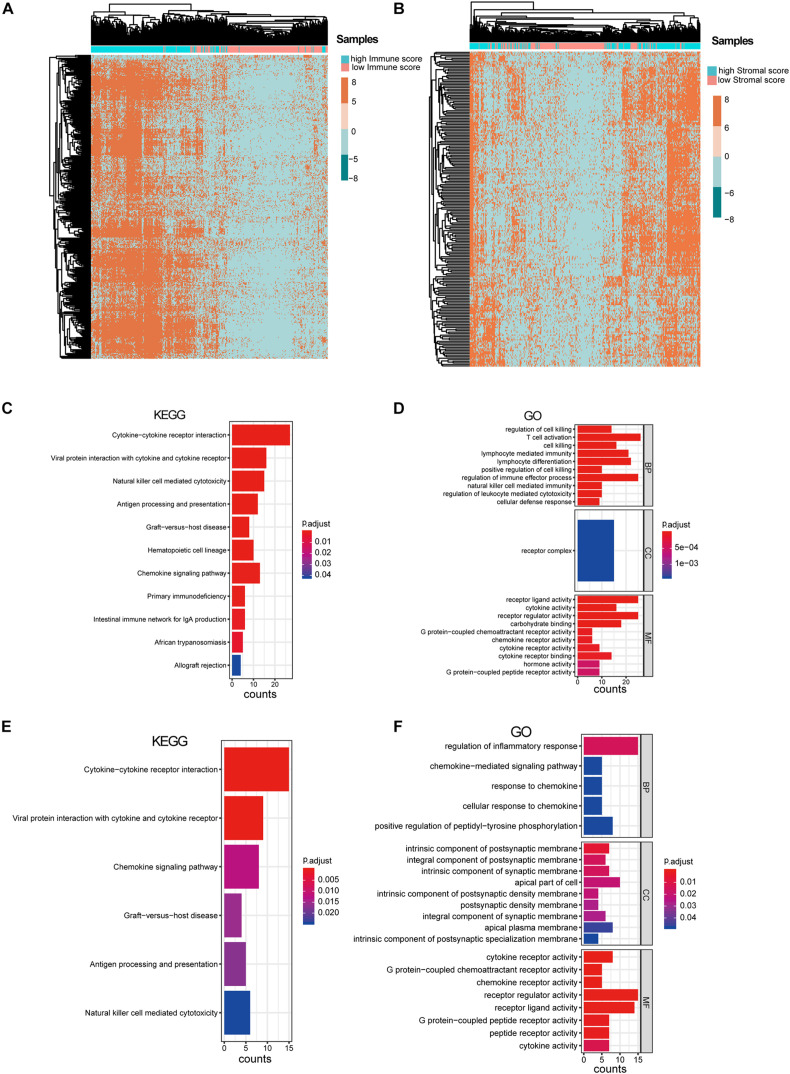
Analysis of immune-associated differentially expressed genes (DEGs) and stromal-associated DEGs. **(A)** Heat map of immune-associated DEGs in skin cutaneous melanoma (SKCM) samples. **(B)** Heat map of stromal-associated DEG genes in SKCM samples. It shows that only immune-associated DEGs could nicely separate the low- vs. high-immune-score samples. **(C,D)** Enrichment analysis of the immune-associated DEGs. **(E,F)** Enrichment analysis of the stromal-associated DEGs.

After excluding the patients with no survival information, 429 qualified patients of the TCGA SKCM dataset were selected. Corresponding clinical traits that include overall survival information were also downloaded. Based on the ESTIMATE analysis results, we divided the SKCM patients into high- and low-immune-score or high- and low-stromal-score groups. Then, we identified DEGs between these high- and low-score groups. According to the immune scores, 321 genes were differentially expressed, including 316 up-regulated genes and five down-regulated genes (Figure 1B). Similarly, there were 205 DEGs based on stromal scores; interestingly, all of them are up-regulated (Figure 1C). We found no intersection between these immune-related DEGs and the stromal-related DEGs. It suggested that the two types of DEGs performed different functions in SKCM. Then, the heat maps hinted at the gene expression patterns of DEGs (Figures 2A,B), and it was found that the immune-related DEGs had better classification efficiency. Then, the enrichment results showed that the immune-related DEGs were mainly enriched in the chemokine signaling pathway, cytokine–cytokine receptor interaction, primary immunodeficiency (KEGG) (Figure 2C), lymphocyte-mediated immunity, T cell activation, and regulation of immune effector processes (GO terms) (Figure 2D). It showed that the results of the ESTIMATE analysis are credible. The stromal DEGs were mainly enriched in cytokine–cytokine receptor interactions, antigen processing and presentation, natural killer cell-mediated cytotoxicity (KEGG) (Figure 2E), regulation of inflammatory response, and cellular response to chemokine (GO terms) (Figure 2F). These results indicate that the DEGs we screened are closely related to the immune response in SKCM patients, which may be used as new biomarkers for SKCM. Because the immune-related DEGs had a better classification efficiency by the cluster analysis, we use the 321 immune-related DEGs for further analysis.

### Identification of Gene Co-expression Modules That Associated With Clinical Traits

**FIGURE 3:**
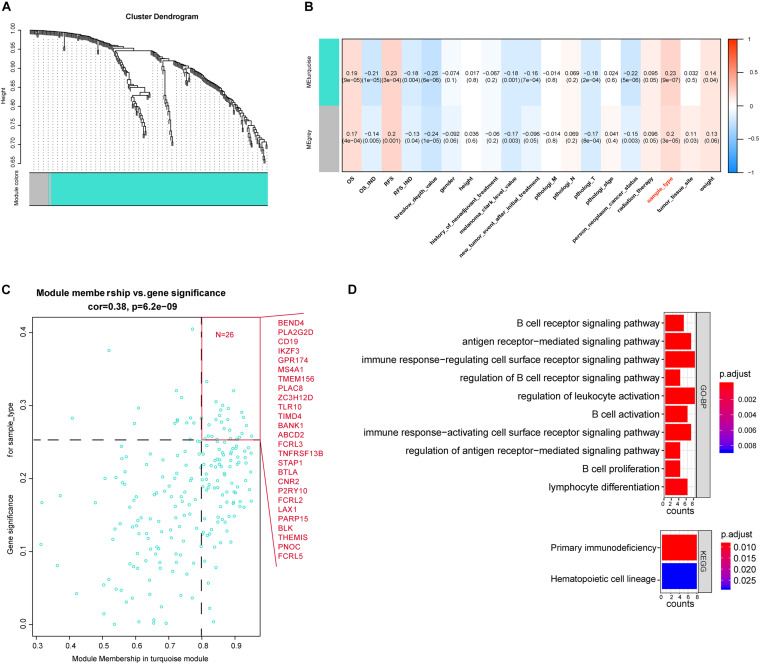
Weighted gene co-expression network analysis (WGCNA) of immune-associated DEGs. **(A)** Cluster plot of genes based on the topological analysis of WGCNA. It shows that these immune-associated DEGs had two expression patterns. **(B)** Relationships between the module and clinical traits. Each cell described the relationship coefficients and *P*-values. **(C)** The gene significance and module membership of the blue module associated with metastasis. Hub genes are shown in red front. **(D)** Kyoto Encyclopedia of Genes and Genomes and Gene Ontology analysis of metastasis-associated hub genes.

After differential analyses, we selected the 321 immune-related DEGs to build the gene co-expression network by WGCNA. The cutoff of soft power was set at 10 because it could make the scale-free topology model fit *R*^2^ reach 0.85, and the mean connectivity is less than 20. This indicates that we have built a scale-free network (Supplementary Figures 1A–D). Then, we set the minimum module size at 30 to filter the co-expression modules. Finally, turquoise and gray co-expression modules were built (Figure 3A). The heat map described the topological overlap matrix (TOM) of input genes and showed the relationship between the two modules (Supplementary Figure 1E). The results showed that the 321 immune-related DEGs were expressed in two patterns.

### Identification of Crucial Modules

We calculated the relationship between the two modules and clinical traits (Supplementary Figures 1E,F) and then selected the essential genes. The results showed that the turquoise module, which contains 219 genes, was significantly associated with sample type (Figure 3B). Sample type stands for the primary tumor or the metastatic one. Based on the cutoff (GS > 0.25 and MM > 0.8), we identified 26 crucial genes out of the 219 turquoise module genes (Figure 3C). The enrichment result of the 26 genes showed that they were enriched in the primary immunodeficiency pathway and immune cell-associated signaling pathways. It suggested that these genes may play a crucial role in the metastasis of SKCM (Figure 3D).

### Validation of the Crucial Candidate Genes

**FIGURE 4:**
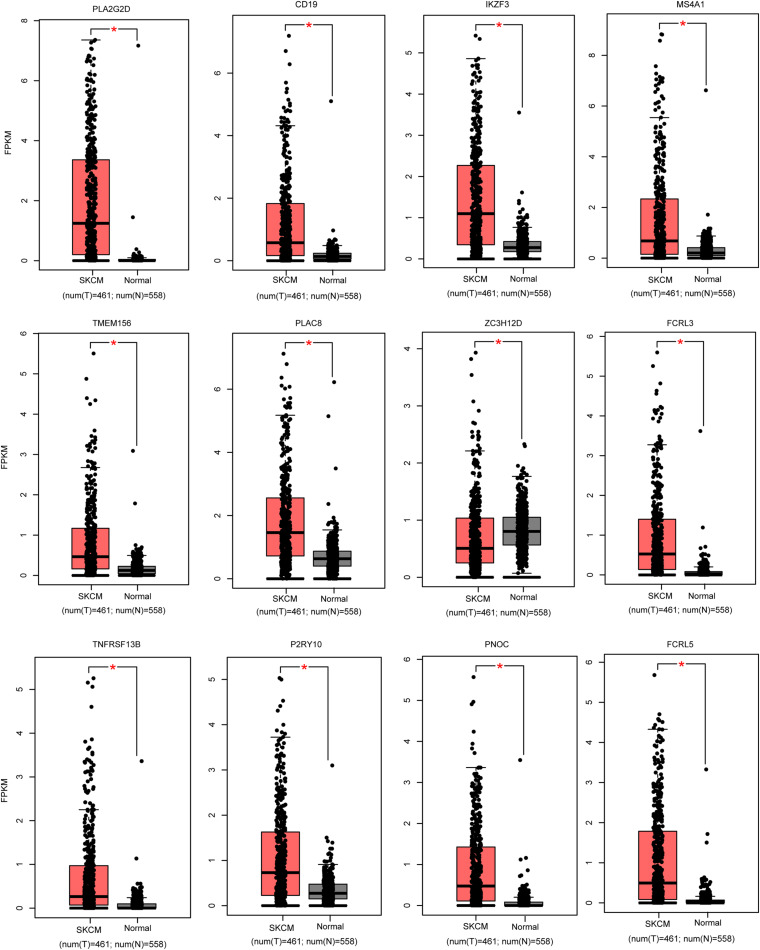
Validation of the expression pattern of hub genes in melanoma. The red color represents the melanoma samples, while black indicates normal samples. *P < 0.05.

We used the GEPIA (see text footnote 1) database to screen the 26 candidate DEGs that were not only immune-related DEGs but also differentially expressed between SKCM patients and normal samples. This screening procedure can help us obtain the biomarkers with more potential for clinical application. Finally, we obtained 12 crucial genes that are differentially expressed in cancer patients compared with normal samples and correlated with the tumor immune microenvironment (Figure 4 and Table 2).

**TABLE 2:** Basic information of the 12 crucial genes.

Gene symbol	Full title	Module membership in turquoise module	Gene significance	*P*-value of differential analysis	
				High immune score vs. Low immune score	*SKCM* vs. *normal*
PLA2G2D	Phospholipase A2 group IID	0.896203345	0.320391963	2.74E–51	9.16E–64
CD19	CD19 molecule	0.842601708	0.305343554	5.57E–35	5.81E–40
IKZF3	IKAROS family zinc finger 3	0.862856409	0.300451054	6.19E–44	9.03E–52
MS4A1	Membrane Spanning 4-Domains A1	0.857009326	0.294092449	3.77E–36	3.12E–32
TMEM156	Transmembrane Protein 156	0.920464075	0.290862446	5.28E–55	7.99E–40
PLAC8	Placenta associated 8	0.853632449	0.286478015	3.81E–45	2.47E–43
ZC3H12D	Zinc finger CCCH-type containing 12D	0.926001735	0.283865057	2.74E–58	0.0151
FCRL3	Fc receptor like 3	0.934510176	0.272729855	2.08E–51	5.46E–46
TNFRSF13B	TNF receptor superfamily member 13B	0.867106521	0.268605275	1.70E–43	7.14E–32
P2RY10	P2Y receptor family member 10	0.950493884	0.258242908	5.55E–55	2.55E–36
PNOC	Prepronociceptin	0.845668597	0.253861151	2.65E–38	2.06E–48
FCRL5	Fc receptor like 5	0.846566936	0.250761762	1.12E–37	3.66E–47

### The Crucial Genes are Potential Prognostic Biomarkers

**FIGURE 5:**
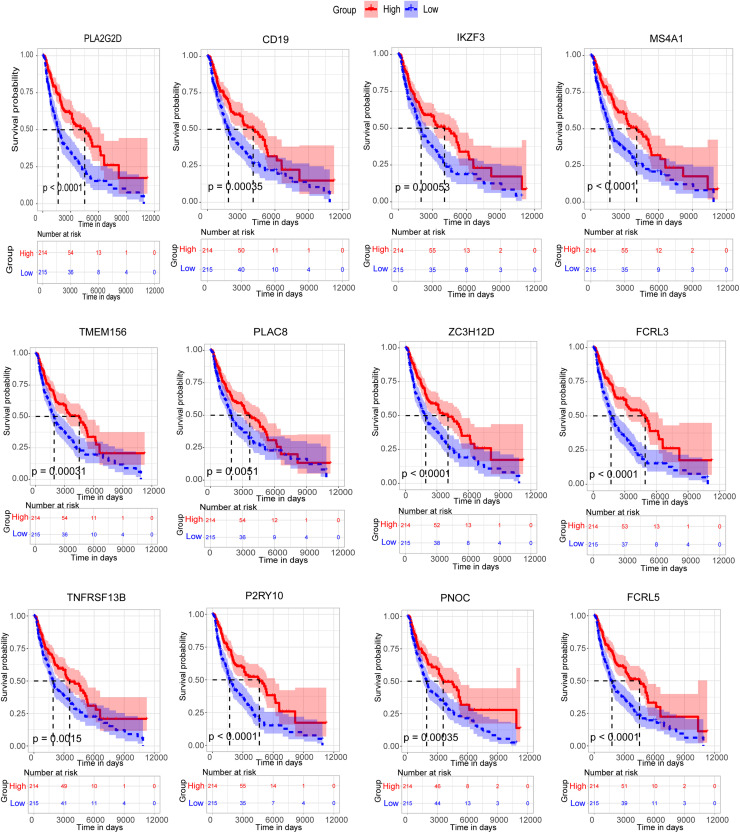
Survival analysis of 12 candidate metastasis-associated key genes.

**FIGURE 6:**
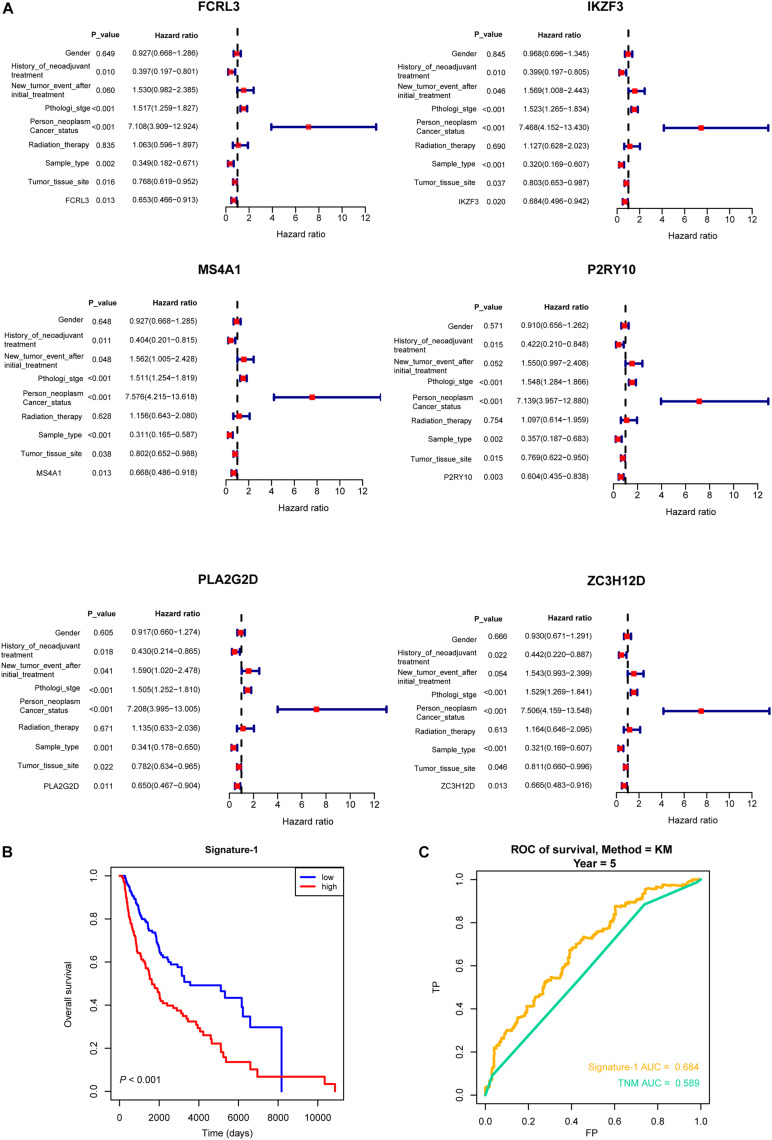
Multivariable Cox regression analysis of crucial genes. **(A)** Forest plot showing the six hub genes (PLA2G2D, IKZF3, MS4A1, ZC3H12D, FCRL3, and P2RY10) that were independent prognostic factors in skin cutaneous melanoma. **(B)** The survival model (Signature-1) constructed by the six genes and the Kaplan–Meier curve which showed that it was survival-associated. **(C)** Receiver operating characteristic analysis showing that Signature-1 performed a better prognostic efficacy than the TNM stage.

Then, all the 12 essential genes (PLA2G2D, IKZF3, FCRL3, FCRL5, PNOC, PLAC8, P2RY10, TMEM156, ZC3H12D, MS4A1, CD19, and TNFRSF13B) were tested by survival analysis. We divided the patients into high- or low-expression groups based on the median expression level of the genes and performed a survival test. It found that all of them have a good prognostic efficacy in SKCM (survival *P* < 0.05). Interestingly, all the 12 genes are protective factors (Figure 5). A test dataset including 1,085 SKCM patients in the OSskcm Tool also testified the prognostic ability of these candidate genes (Supplementary Figure 2A). Then, we performed a multivariable Cox regression analysis and found that six of these genes (PLA2G2D, IKZF3, MS4A1, ZC3H12D, FCRL3, and P2RY10) were independent prognostic signatures (Figure 6A). Next, we combined these genes into a Cox proportional hazard model to construct a survival signature (Signature-1). We explored the survival efficiency of Signature-1 (*p* < 0.05, Figure 6B). Then, an ROC analysis was used to compare the prognostic value between Signature-1 and TNM stage, and we found that Signature-1 had a better prognostic efficacy than the TNM stage (Figure 6C). Next, the test dataset that contained 54 SKCM patients was used to verify the efficacy of the signature, and the result showed that Signature-1 kept its prognostic value in the test dataset (Supplementary Figure 2B). All the results hinted that the six genes had an excellent prognostic efficacy in SKCM, and we developed a survival model associated with tumor microenvironment and metastasis which may be applied to the clinic.

## Discussion

Thousands of people worldwide suffer melanoma every year, and the number of SKCM is growing faster than any other type of malignancy. The numbers of research demonstrate the role of the immune cells on tumor cells, and the immune components in melanoma tissue can be used to evaluate the therapeutic and prognostic efficacy in melanoma (Ladanyi, 2015). Patients with primary tumors usually have higher than a 5-year survival rate (Balch et al., 2009). Bioinformatics analysis is widely used in the discovery of various biomarkers (Chen et al., 2020). Thus, obtaining predictive biomarkers for prognosis has become a priority.

WGCNA is an algorithm used to find crucial modules from a gene expression (Luo et al., 2019). Candidate therapeutic biomarkers are identified based on the relationship between the modules and the phenotype. Here we constructed the co-expression modules *via* WGCNA using the DEGs in high-immune-score SKCM patients compared with low-immune-score SKCM patients. Then, we obtained 12 crucial genes associated with the metastasis of SKCM, and six of them were independent prognostic biomarkers. The survival model of the six genes had a good predictive efficacy. We also used the TIMER web to verify the association between the six genes and immune cells (Supplementary Figure 3); all of them are associated with immune cell infiltrate levels. In addition, FCRL3 can promote IL-10 expression in B cells through the SHP-1 and p38 MAPK signaling pathways and is highly expressed on CD4 + CD26- T cells (Wysocka et al., 2014; Cui et al., 2020). IKZF3 is a predictor for survival in multiple myeloma stage III patients (Awwad et al., 2018). MS4A1 is associated with apoptosis of B-cell lymphoma Ramos cells (Kawabata et al., 2013). P2RY10 has been reported to be a tumor microenvironment-associated gene and a potential diagnostic biomarker of metastatic melanoma (Wang et al., 2018, 2020). PLA2G2D has been reported to moderate inflammation and could be a potential biomarker for treating inflammatory disorders (Miki et al., 2013). ZC3H12D is associated with inflammation (Huang et al., 2018). In SKCM, we first found that these crucial genes are involved in metastasis and perform similar functions in our WGCNA network. At the same time, they have a good prognostic efficacy. All of these genes have potential clinical applications as key prognostic biomarkers.

All in all, our findings may improve our fundamental knowledge of the molecular mechanisms of SKCM, and these prognostic biomarkers may improve the treatment of this cancer.

## Conclusion

Firstly, we filtered the immune-associated DEGs by the ESTIMATE analysis and got a metastasis-associated module through WGCNA. We then obtained overlapping DEGs in SKCM patients compared with normal samples and in the immune microenvironment, and 12 genes were screened. Next, we used survival analysis to obtain crucial prognostic biomarkers, and six genes with independent prognostic efficacy were filtered. The results may be helpful for future studies concerning SKCM to find potential prognostic targets.

## Data Availability Statement

The original contributions presented in the study are included in the article/Supplementary Material, further inquiries can be directed to the corresponding author/s.

## Author Contributions

YL, SL, and SH conceived and devised the study. YL and SL performed the bioinformatic and statistical analysis. ZG, WZ, PW, and YS found related data and analysis tools. YL, JH, and SH supervised the research and wrote the manuscript. All authors contributed to the article and approved the submitted version.

## Conflict of Interest

The authors declare that the research was conducted in the absence of any commercial or financial relationships that could be construed as a potential conflict of interest.

## Footnotes

1 http://gepia.cancerpku.cn/

## Supplementary Material

Supplementary Figure 1 Weighted gene co-expression network analysis (WGCNA) in the study. **(A,B)** Topology of the co-expression network. **(C,D)** Scale-free topology based on the cutoff of power (power = 10). **(E)** Visualization of the WGCNA network. Heat map showing the TOM among all modules. **(F)** The clinical trait information of 429 skin cutaneous melanoma patients.

Supplementary Figure 2 Test datasets were used to present the prognostic efficacy of six crucial genes. **(A)** Survival analysis of six crucial genes in a dataset of 1,085 SKCM patients. **(B)** A test dataset used to show the prognostic efficacy of Signature-1.

Supplementary Figure 3 Correlation of six hub genes with immune infiltration in melanoma.
