# Supplementary figures and images for: Identification of Potential Prognostic Biomarkers Associated With Cancerometastasis in Skin Cutaneous Melanoma
Source: Front Genet. 2021 Jul 21;12:687979. doi: 10.3389/fgene.2021.687979 (PMC8337057; doi:10.3389/fgene.2021.687979)

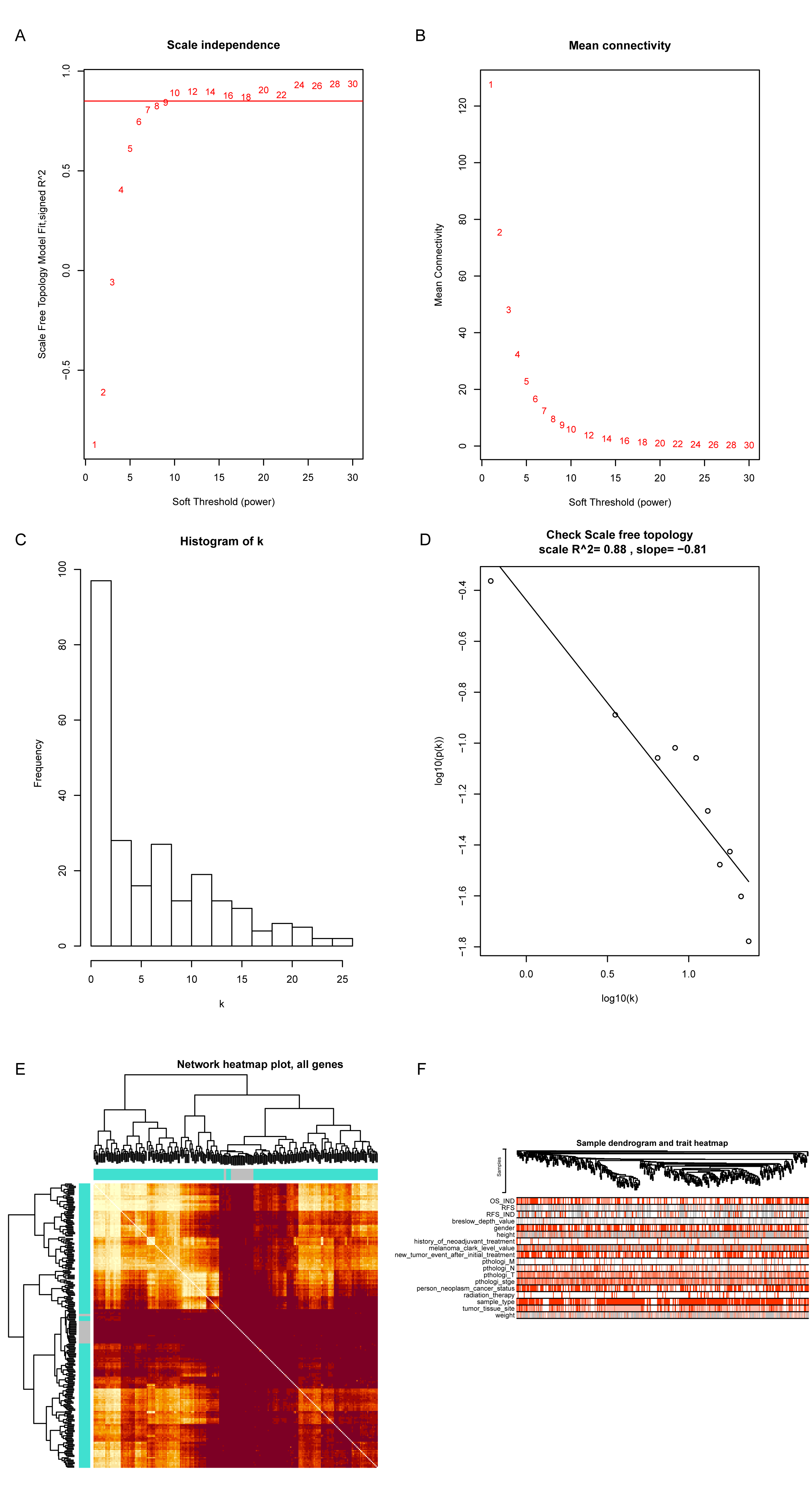

Supplement: Supplementary Figure 1 — Weighted gene co-expression network analysis (WGCNA) in the study. (A,B) Topology of the co-expression network. (C,D) Scale-free topology based on the cutoff of power (power = 10). (E) Visualization of the WGCNA network. Heat map showing the TOM among all modules. (F) The clinical trait information of 429 skin cutaneous melanoma patients. [file Image_1.TIF]

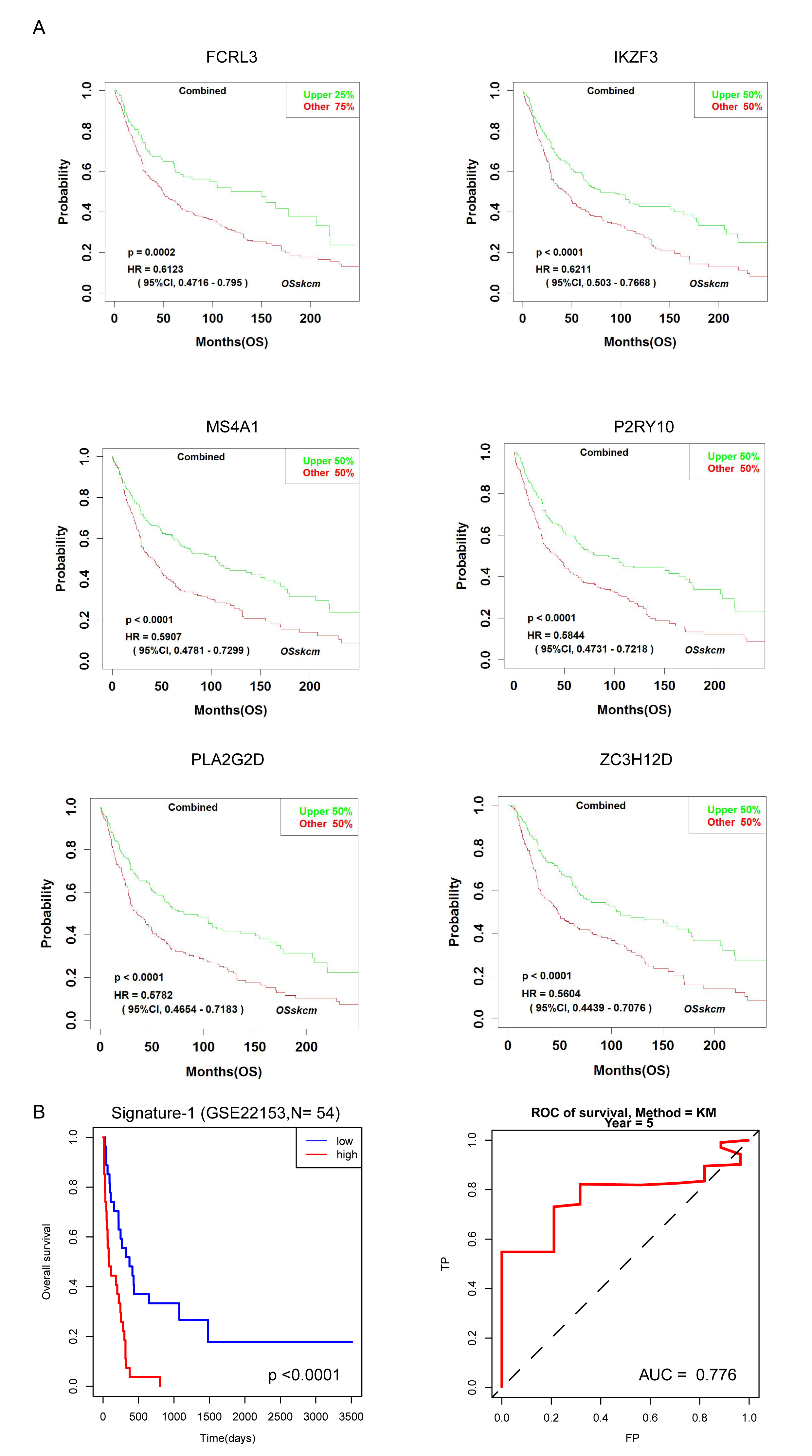

Supplement: Supplementary Figure 2 — Test datasets were used to present the prognostic efficacy of six crucial genes. (A) Survival analysis of six crucial genes in a dataset of 1,085 SKCM patients. (B) A test dataset used to show the prognostic efficacy of Signature-1. [file Image_2.TIF]

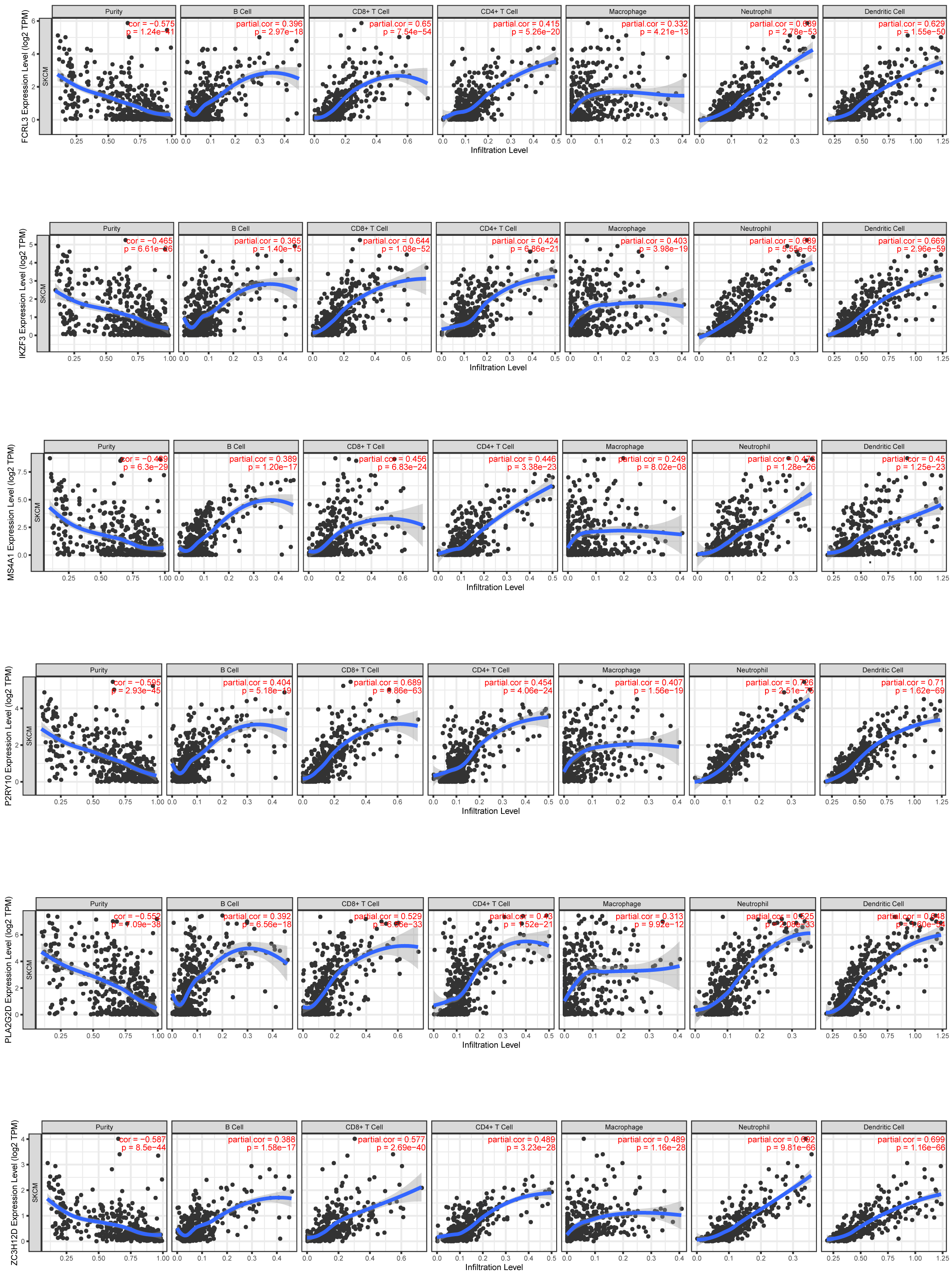

Supplement: Supplementary Figure 3 — Correlation of six hub genes with immune infiltration in melanoma. [file Image_3.TIF]
